# Mechanical Abrasion of the Teeth

**Published:** 1893-03

**Authors:** Junius E. Cravens

**Affiliations:** Indianapolis, Ind.


ARTICLE III.

### MECHANICAL ABRASION OF THE TEETH.

BY JUNIUS E. CRAVENS, D. D. S., INDIANAPOLIS, IND.

Read before the Ohio State Dental Society, December, 1892.

After completion, the crowns of teeth are subject to such modifications only as are destructive to contour. Aside from decay, these modifications are usually known as abrasions, and are classed as mechanical and chemical, so called. This paper is devoted to a study of mechanical abrasion, because it is observable to some extent on every set of adult teeth that are in occlusion. Mechanical abrasion should not be confounded with conditions arising from blows, violence of sudden occlusion, as in snapping the teeth, or any of those accidental coincidences that crack and break away fragments of tooth-crowns. All modifications that should be classed as simply abrasions are accomplished by a gradual wearing away of the hard tissues of the crown, whether classed as mechanical or chemical abrasion. Usually, mechanical abrasion is confined to those parts of a crown that are, or have been, in occlusion with teeth of the opposing maxillary. Mechanical abrasion is confined to those parts of a crown that are, or have been, in occlusion with teeth of the opposing maxillary. Mechanical abrasion is essentially progressive, and presents three well-marked stages for consideration—each having characteristics that are well worth the attention of the practitioner.

### PRIMARY ABRASION.

The primary stage is confined to the enamel, which is worn off in small, flat and highly polished surfaces, consequently, in this stage, the abraded tracts are colorless and without sensibility.

Careful observation of cases as they occur in daily practice has led the writer to a recognition of the following propositions:

First: The primary stage of mechanical abrasion is the accomplishment of complete occlusion; and—

Second: Either condition is impossible without the other; therefore—

Third: The primary stage of mechanical abrasion is not detrimental to the use or tenure of teeth; to the contrary—

Fourth: It is essential to a proper and most effective exercise of the teeth in mastication.

It is much to be regretted that the process of mechanical wear of the teeth does not cease with the establishment of the primary stage.

When a tooth is erupted, it is then perfect—if ever perfect—in contour, and to be perfect in contour means to present certain angles and curves that render impossible that adjustment that must be mutual between teeth—that we style complete articulation—without some abrasion of the occluding surfaces. The practical impossibility of frequent, forcible contract of equally hard substances, without some abrasion of both, must always be considered in mechanical adjustments that are designed to be persistent. Dentists who fail to recognize and provide against possible destructive force of occlusion, primarily—emphasized by inevitable mechanical abrasion, secondarily—are certain to experience a liberal per cent. of failures in their operations.

SECONDARY ABRASIONS.

The secondary stage is produced by a continuation of the wear that begins with the first stage; but in the secondary the abraded tracts are observed to have notably broadened, and are depressed centrally so as to appear as distinct saucer shaped facets, sometimes attaining great depth; they are usually discolored—varying from yellow to a coffee-brown. It is characteristic of animal ivory, from whatever source, to become discolored by exposure to air and moisture, and general surface contamination; in abraded tracts on teeth it is observed that the deeper the discoloration the less the degree of sensibility; however, their effect often is only superficial.

When abrasion has progressed until the cusps have been reduced to their bases, the tracts of exposed dentine hang like sagging canvass from the enamel that has been left standing as an elevated rim around the exposure, as if for protection. This elevation of rims of enamel about abraded tracts constitutes a distinguishing and important feature of advanced abrasion, and should be noted.

Unlike the primary stage, the second involves two of the hard tissues of the crown in its destructive progress; in no sense is it essential to complete articulation of teeth; in fact, it often destroys established articulation and continues without discernable contact. However, the secondary stage, although undeniably erratic, marks a degree of progress in a transition by which man, in decline of life, comes to use his masticators much after the fashion of a goat.

Secondary abrasion is not altogether without redeeming features; it certainly admits of a more liberal lateral action of the jaw; facilitates prehension and accommodates retraction in occlusion. Further, as this stage does not obtain until the individual is nearly or quite fifty years old, it is largely instrumental, by virtue of a shortening of the visage, in giving to the features that expression of manhood or womanhood that so often is an index to the character, and that fixes itself indelibly upon us for good or evil, until the softening snows of the winter of life come to take away the settled look of business and cares; and then the man is old.

TERTIARY ABRASION.

In the third stage of mechanical abrasion, we discover the extremely worn-out condition of the teeth, so characteristic of old age, or, at least, beyond middle life. In many instances that which usually should be an articular surface, has been reduced to a single deep depression, touching—if at all—at the sides only; this is often observed in the cuspids and incisors. In many cases of abrasion of the molars there remains of the grinding portion, the vertical rim surrounding the abraded tract and standing up in relief, while the central portion is occupied by an irregular ridge of enamel—the remaining evidence of grinding surface fissures. Here we have a practical completion of the transition referred to in closing observations on the secondary stage, by which man in the "sear and yellow leaf" is relegated to the herbivora; let us hope he may not become quite an ass. With the angle of his jaw nearly straightened, his teeth notably shortened by abrasion, the cusps of his youth worn entirely away, and circular and traverse ridges of enamel rising like those of the horse to perform the manual of mastication, we observe the "lean and slippered pantaloon" comminuting his food into meal with his teeth, instead of bruising and triturating it in saliva as in the days of his cusps; that which he can not chew he eschews, so that gradually he accommodates himself to a diet in which slops, eggs and tender vegetables predominate or dominate.

To the aged it is a blessing that they are enabled to manipulate a worn-out mechanism of mastication so effectually as to enjoy fair digestion. Thus we come to comprehend that although the tertiary abrasion is so disastrous to the teeth themselves, it renders perhaps reasonable compensation in enabling the sharp edges and ridges of enamel to meet the deficiencies of muscular weakness and impaired digestive power.

Again, in the third stage, it becomes a matter for regret that the progress of wear of tissue may not be stayed; but the fact of continual wear here is as merciless as in the preceding stages; the crowns disappear, and the subject continues to chew—or thinks he does—after a fashion, with such poor stumps as may have been spared, until at last all are gone, and the edentulous gum of infancy is almost reproduced in "second childhood." Still the compensating kindliness of Nature is asserted, and the gums become so toughened as to prove effective in bruising such articles of d et as are appropriate to that stage of existence; an intimacy of the nose and chin is threatened, and the countenance of the individual whose "sands of life" are run so low, assumes the angelic expression of one who has realized the hollowness of life's ambitions and appetites, and fixes an expectant gaze upon eternity.

The density of enamel enables it to offer a greater resistance to abrasion than dentine is able to do, otherwise the wear would be uniform, and the teeth would become flat and smooth, offering no advantages as grinders. The dentist should consider well the consequences to the patient in his hands, ere he proceeds to level up the saucer-shaped depressions in the grinding aspect of molars and bicuspids of the middle-aged or aged; likewise there is here a suggestion to be applied to prosthetic practice.

### AGE ESTIMATED BY ABRASION OF TEETH.

In most cases the approximate age of a subject may be estimated by the stage of abrasion of the teeth. The first stage (enamel wear) is found as early as eighteen years, and even earlier. The second stage should be well marked at fifty, and often noted by forty years. The third stage (extreme wear) is nearly always apparent at sixty years. Habit often has much to do with abrasion, and an individual's teeth may thus be made to indicate much greater or less age than the fact.

Whether a problem is presented herein, or not, depends upon the "point of view," and some other things. —*Cental Register.*

